# The case of the ‘Spurious Drugs Kingpin’: shifting pills in Chennai, India

**DOI:** 10.1080/09581596.2019.1593948

**Published:** 2019-06-04

**Authors:** Sarah Hodges

**Affiliations:** Department of History, University of Warwick, Coventry, UK

**Keywords:** India, pharmaceuticals, Chennai, fake, pseudo, global health

## Abstract

This paper recounts the tale of a ‘Spurious Drugs Kingpin’ and his scandalous business empire of relabelling and recirculating expired medicines in the south Indian city of Chennai. At the time the story broke, in the first half of 2010, questions of drug safety dominated media headlines and public discussion. However, a closer investigation suggests a more complicated picture. While relabelling expired medicines was certainly a crime, it is far from clear if their relabelling and subsequent redistribution constituted a public health danger. If actually existing unsafe drugs did not fuel this drug safety scandal, what did? I argue this case illuminates two things: (1) the illusory nature of certainties about drug safety and (2) how ambiguity about the safety of expired drugs facilitates policing not of relations within the pharmacological world, but instead of social and economic relations. This episode matters because it illuminates how, within apparent attempts to police the safety of circulation of drugs, drugs themselves are neither subject nor object, but instead the ground upon which market battles are waged.

## Anatomy of a scandal

Chennai, the capital of the south Indian state of Tamil Nadu, is a city thick with health care. Moving around the network of roads that knits the city together, it often feels like some variety of hospital sits on each and every street corner. Patients, or their family members, hurry from their homes, small hospitals, and nursing facilities to the more than 42,000 pharmacies across the state, where they buy physician-prescribed and over-the-counter medicines. Others buy their medicines from in-house pharmacies run by so many of the private hospital chains. Drawing patients from near and far, and long-renowned for its high quality of health care (Hodges, 2016), Chennai gets to work looking after those who are sick.

So in March 2010, when the news broke that a three-year-old girl had died a few months earlier after taking ‘antibiotics’ that her parents had bought from a pharmacy to treat her fever, the media picked up the story and the scandal gripped the city for weeks (“CM’s strong dose to fake drug units,” 2010; “Timeline,” 2010). Newspaper headlines and radio and television news outlets broadcast the bad news every day for more than a month: spurious (or ‘fraudulent’) drugs had found their way into Chennai’s pharmacies. In response, city police carried out raids of homes and offices. Evidence mounted. And any early attention paid to the death of a little girl was overshadowed by updates on the on-going search for the culprits. Early on, four lakh rupees’ worth (about $9000) of suspect drugs were seized.^1^ The police declared that they were on a ‘war footing’ as they carried out their search for suspects (Mallady, 2010).

Before long, newspapers reported that the criminal masterminds were neither retail pharmacists nor drug manufacturers. Instead, the police pointed to a network of distributors. By the end of May 2010, the police had 12 people in custody. They were charged with forgery and cheating offenses (“Drug Company Owner Surrenders,” 2010). The 12 included the man who was dubbed the ‘Spurious Drugs Kingpin’ by the city’s media and who was to become the ‘chief accused’ in the case.

Distributors are a largely invisible linchpin that connects consumers to drugs. They move drugs from where they are manufactured to the retail outlets where they can be bought. Newspaper articles alleged that the culprits, having properly collected expired, or nearly expired, drugs from retailers, deviated from the law. As the story unfolded, readers were given a picture of a sophisticated racket that precisely exploited the strict regulations that exist to protect consumers from purchasing expired drugs.

Under the terms of India’s Drugs and Cosmetics (Amendment) Act 2008, pharmaceutical retailers are incentivized keep a close eye on the expiry dates printed on the packaging for the products that they stock. All retailers are required to keep a box labelled ‘NOT FOR SALE’, in which they store about-to-expire products. To guarantee that retailers incur no loss for unsold, expired stock, the law mandates that manufacturers must arrange for the regular collection and disposal of these items (Kannan & Hiddleston, 2010). Just as distributors bring drugs from the manufacturer to the retailer, they also tread the circuit in reverse, collecting unsold drugs from retailers. However, in this case, rather than disposing of these drugs as required, distributors re-labelled the drugs with false expiry dates. They then redistributed these relabelled drugs, having illegally imprinted them with longer shelf lives.

At the time the story broke, the urgency in the reporting turned on the immediate dangers that these drugs presented to the consuming public. However, a closer investigation of this episode suggests a more complicated picture. Officials at the state’s Drug Control Authority were quick to reassure members of the public that, despite the tenor of the news coverage, none of the expired drugs identified had reached pharmacies or been sold to consumers. Indeed, even the state’s Chief Minister sought to calm the restive Legislative Assembly by reminding the elected members of the distinction between expired drugs and poison (‘Expired drugs safer,’ 2010). Few believed them, and one state legislator suggested that the sellers of the drugs in question should be punished by the same laws that are used for the crime of ‘murder for gain’ (‘Who stands where,’ 2010). In the febrile environment, one seemingly vital element in the story remained significantly under-reported. Whilst the distributors’ alleged actions were certainly *illegal*, as the Chief Minister’s comment attempted to underscore, there was never any direct evidence put on display to show that these redistributed drugs were in fact *dangerous*. Revisiting this scandal thus presents a puzzle: If the public was not necessarily in danger, then what, exactly, was all the hue and cry about?

There is complicated evidence on the dangers of expired drugs (Tull, 2018). Many see them as a kind of ‘least-worst’ option in resource-constrained settings. Despite Indian pharmaceutical manufacturers being popularly referred to as the ‘pharmacy to the third world’, Indian pharmaceuticals – particularly when circulating in African markets – are also regularly cast as suspect objects (McLaughlin, 2012). Recent history shows India’s preoccupation with regulatory stringency regarding drugs quality, for example refusing humanitarian donations of expired drugs in the wake of a devastating earthquake in 2001 (Tull, 2018). The peculiar temporality of expired drugs thus presents an excellent example of how ambiguity, rather than certainty, can underscore the cut-and-dried safety advice that we have come to expect from public health policy and practice.

India’s southern state of Tamil Nadu, where this story unfolded, presents another level of complexity. On the one hand, the state has for decades been lauded as a development leader within India, boasting of some of the country’s best health indicators and being regularly singled for efficiency in state government and health care delivery. On the other hand, the state is also home to a buoyant private health sector; corporate health care managers explain that the market conditions are ideal, and that health care in Tamil Nadu is seen by private investors as a ‘recession-proof industry’ (Hodges, 2013). Statistics reveal a great deal of morbidity, but not extraordinary mortality; people are poor, but not too poor to be willing to pay for repeated treatments.

## The suspects

Over the course of the media coverage of April and May 2010, individual characters emerged along with the details of the scam.

One such suspect, Venkatesan, worked as a van driver for the drug distribution company in question. Soon after being named by the media, Venkatesan surrendered to the police and detailed his practices in official statements to the authorities. These, in turn, fuelled media reports; readers learned that it was Venkatesan who did the distributors’ work of collecting the expired, or nearly expired, contents of the pharmacists’ NOT FOR SALE boxes. But instead of disposing them at the city dump, as required by law, Venkatesan delivered them to a godown (warehouse) (Sujatha, 2010).

It turned out that Venkatesan was from Kodungaiyur, a district in the far north of Chennai that houses the city’s largest and oldest municipal dump, the exact dump where the drugs were meant to be disposed (Mallady, 2010). And within Kodungaiyur, he was from the Ezhil Nagar neighbourhood, a tightly packed and mainly residential area abutting the vast dump. Houses repurposed as small workshops sit among the modest, if *pukka* [brick, rather than mud], homes that line its narrow lanes. These include many businesses where workers sit alongside open gunnysacks full of recovered scrap, sorting the materials into different categories; these objects in turn fuel the area’s many small manufacturing businesses that turn the city’s discards into sellable items (Hodges, 2013). Small vans like the one Venkatesan drove regularly move in and out of the area, collecting or discharging stock.

Enter Ravi. Ravi also hailed from Ezhil Nagar.

Whereas Venkatesan collected the expired drugs, it was Ravi to whom Venkatesan delivered them. Ravi then transported the drugs from Ezhil Nagar to Koyambedu (Mallady, 2010). Koyambedu is on the western fringe of the city and is home to both the city’s largest fruit and vegetable market as well as the city’s long-distance bus terminus. It is a dense transfer point for goods and people, and it is where the drugs relabelling operation was located. In Koyambedu, newspapers reported, there was a residential house, surrounded by a small walled compound where workers repurposed the expired drugs, using chemicals to remove the manufacturers’ expiry and batch dates printed on the foil, and then reprinting the packets with fresh dates. These drugs would then be distributed to new retailers (Kannan & Hiddleston, 2010). According to Venkatesan’s statements to the police, not only did the entire relabelling operation take place at the business premises of a legitimate licensed distributor for the drugs – Meena Medical Agencies – owned by Meenakshi Sundaram, it was masterminded by him, too.

## The importance of being meenakshi sundaram, Spurious Drugs Kingpin

The burden of scandal fell unevenly.

Meenakshi Sundaram owned and operated the drugs distribution company that emerged at the centre of the scandal, and he had gone on the run. In the following weeks’ media coverage, it emerged that the police seized Rs1 (c $210,000)crore worth of drugs from the Kingpin’s storage facilities.

Meenakshi Sundaram was already well known to the police (“Key Accused Surrenders,” 2010). This 2010 episode was the third time in as many years that he had been sought in relation to distributing spurious drugs (Vannan, 2010a). Earlier investigations suggested that he had been distributing relabelled drugs to pharmacies in Tirunelveli, nearly 400 miles south of Chennai (Vannan, 2010b).

Meenakshi Sundaram had swagger. Newspapers wrote lyrically about his life as a man-about-town:

From riding a moped to driving into the city’s Cosmopolitan Club in a Ford Endeavour SUV, Meenakshi Sundaram, whose present worth is estimated to be over Rs 100 crore,^2^ has come a long way in 17 years. … He owned three cars and struck deals with other businessmen at the Cosmopolitan Club on Anna Salai [the city’s most established shopping district], where he was known to spend his evenings in the company of affluent and elite friends. Sundaram was conscious of his looks and made extra effort to groom himself. He was dressed immaculately and lived in style. In his two flats in Chinmayanagar [an affluent neighbourhood in the west of Chennai], the police said, ‘Huge framed photographs of him adorn the walls of the posh apartments that have a wide collection of luxurious goods. … His children go to elite schools’ (Vannan, 2010a).

Meenakshi Sundaram had financing. He ‘operated eight bank accounts’ (The Hindu 2010).

Meenakshi Sundaram was the object of outrage. This included among officers of the court (judges, attorneys, bailiffs, clerks). Newspapers reported:

In a shocking incident a group of about 20 advocates manhandled persons accused in a recently revealed case of a flourishing spurious drugs trade, and called aloud for their ‘hanging’, when they were brought to the Egmore court on Wednesday, created a tense situation for about half an hour. As the policemen escorting Meenakshi Sundaram … to the court played safe and refrained from using force to keep the lawyers at bay, the accused ended up receiving punches and blows to the face and head … and [lawyers] declared that none among them would represent the accused in court (‘Lawyers Play Judge,’ 2010).

Despite that fracas, Meenakshi Sundaram had connections. This repeated record of misbehaviour, without apparent accountability, the papers reported, was due to his substantial financial connections to a major politician in the state (‘Kingpin has Political Links,’ 2010).

***

Most media reporting on policing is structured like a traditional crime mystery, or ‘whodunit’. Police announcements typically take place at specific points in the life cycle of on-going investigations: at the discovery of a crime and the opening of a case, upon bringing charges, at the commencement of a trial. The identity of the mastermind is only revealed upon the completion of a court case. Yet the case of the Spurious Drugs Kingpin was no ‘whodunit’. Readers did not yearn to learn the identity of the Kingpin, we were told at the outset. Instead, readers were pulled in to the story through a daily drip of detail.

Whom did this drip-feed of normally confidential detail serve? While Meenakshi Sundaram’s personal habits and business practices were splashed across the media, there was next to no detail about actual dangers to the public that the expired drugs may – or may not – have presented. Such a state of affairs suggests that it was neither pharmacological safety nor the health of the public that was at the heart of this case. Instead, Chennai’s spurious drugs served as the ground on which other matters were contested.

## Other matters

Just as it seemed that the Kingpin was had secured his leading anti-hero role in this tale, there was a twist. Unsettling questions surfaced in the media’s reporting. What of the state agencies tasked with enforcing the existing regulations designed to keep drugs safe?

Readers also learned that the Kingpin (a distributor), had been identified by manufacturers (his supplier) in successive years leading up to 2010. As one newspaper reported: ‘The [manufacturer], on realizing that “Renerve” tablets with their expiry dates tampered with on the wrapper was being sold in shops in February 2010, drew the attention of drug control authorities’ (‘”Renerve” Exposed Fake Drugs,’ 2010). These manufacturers had urged state agencies to enforce their own regulations.

Another newspaper explained in greater detail:

rumours are already afloat that a pharmaceutical company and the Tamil Nadu Health Department got wind of the [spurious drugs racket] about a year before the complaint was filed with the city police on 16 March. According to a police source, the health department had called a meeting of drug stockists and their distributors, which even alleged kingpin of the racket Meenakshi Sundaram attended, at the Directorate of Medical Services and warned them of such activities. While Health Secretary V K Subburaj denied that such a meeting was held, a police officer gave the low-down of all that transpired behind closed doors. He said it all started when [one manufacturer] found that the sales of its products of certain batch numbers showed a sudden drop in Tamil Nadu. It immediately held its own investigations and found that the decline was due to the circulation of spurious drugs of the company’s products, he added. The company’s officials declined to comment (‘Was Govt Aware,’ 2010).

As media coverage of the scam continued, it came to light that India’s pharmaceutical manufacturers keep very close tabs on their sales figures. In this 2010 episode, a dip in sales alerted the manufacturer that a product was likely no longer on the shelves, despite distributors’ counter-claims that all was business as normal.

Manufacturers have a strong incentive to keep such matters under the radar to maintain both their cash flow and the integrity of their brand name. Newspapers reported earlier instances where manufacturers had realised that the sales of various products had suddenly dipped and, in response, informed the drug control authorities (Vannan, 2010b). These stories suggested that, rather than the police or the state health authorities, manufactures’ concerns about sales figures drove them to attempt to mobilise the state’s drug control agencies to action.

In the Chennai case, however, it seems that manufacturer’s attempts to move the state agencies to act were ineffective; the meeting with the health minister did not achieve the results desired. It is difficult not to speculate that dissatisfaction at this turn of affairs could have led to a Plan B – in the form of a subsequent appeal to the Chennai city police commissioner. If that was the case, it appears that the city police robustly complied with the manufacturer’s request, forming ‘three special teams … to arrest the culprits’ (‘CM’s Strong Dose,’ 2010).

Further muddying the waters, stories abounded in the media that Meenakshi Sundaram had flourished in the pharmaceutical trade thanks to slush money provided by a family member of a senior politician. As one paper reported:

The person who had provided support to him had reportedly called on a senior member of the state Cabinet and requested the transfer of the case to the Central Bureau of Criminal Investigation Department (henceforth, CB-CID) even though the Chennai police have been doing well by arresting or forcing to surrender most of the key players involved in the distribution of spurious drugs (‘Kingpin Has Political Links?’ 2010).

It comes as no surprise, then, that the city police bridled when the Tamil Nadu state government moved to regain control of the case. The Director General of Police (a state office) announced that, given the cross-state nature of the alleged crimes, the case of Meenakshi Sundaram was to be transferred from the city police to the all-India agency, the CB-CID (‘Kingpin Has Political Links?’ 2010). For their part, the city police claimed that it was an attempt to ‘put the lid on a can of worms’ (‘Kingpin Has Political Links?’ 2010).

What finally became of Meenakshi Sundaram? He denied all the charges against him. When he finally faced the court, he did not remain in prison long before he was granted bail. Of the five cases eventually filed against him that have received judgments, no criminal charges stuck (although one unrelated income tax matter was upheld).^3^ His business premises were ordered by the courts to be unsealed.^4^ His license to trade as a pharmaceutical distributor was restored.^5^ He was allowed to distribute the stock in his warehouses.^6^ Of the 12 suspects who were held in connection with the scandal, it is only Meenakshi Sundaram’s name that can be found in the legal record at all, leading one to surmise that all charges against the others were also eventually dropped. Is this, then, the case of the Spurious Drugs Kingpin who got away?

Not according to Meenakshi Sundaram’s legal record, which suggests a man who fought not the administration of justice, but unjust personal persecution. Although it was not granted, Meenakshi Sundaram’s lawyers first filed anticipatory bail applications on his behalf. This is particularly significant because anticipatory bail is a provision in India’s criminal law allows for someone to protect herself from being arrested, in the event false charges are filed against them (typically stemming from personal vendetta and intended only to harass the accused). Meenakshi Sundaram’s lawyers also filed multiple writ petitions on his behalf, a form of legal redress that only India’s High Courts can offer, in the event that an individual’s civil liberties (in Indian legal parlance, ‘fundamental rights’) have been violated.

That is to say, none of the evidence produced in any of the cases against the Kingpin addressed any question of the relative public danger of his alleged crimes. Why not? For the courts, spurious drugs – effectively an Indian legal term for a fraudulent business practice – is about a crime of *theft* rather than a crime of *danger* (Srinivasan, 2010). Yet, in the newspaper coverage and public discussion, the condemnation and the jitters were not in relation to rights being abridged, or fraud in manufacture, but an imminent danger the public. How do we understand the distance between the outrage of the scandal, concerned with life and death, and its legal status that frames the accused as wrongly accused in merely a matter of business?

Spectacles are memorable for the appearance that they create, and in the case of the Spurious Drugs Kingpin, the many newspaper photographs released of uniformed police standing over drugs laid out on tables and ‘perp walks’ produced a story of excess being reined in. In sum, the public scandal that illuminated Meenakshi Sundaram as a culprit and a kingpin, also illuminated the broader public health enforcement bureaucracy and its relationships with the pharmaceutical manufacturing industry, what Hornberger (2018) refers to as the ‘performative world of drug security’. But while these were momentarily illuminated, they remained largely unexplored. The police acted in the name of public safety, but they were protecting the interests of pharmaceutical manufacturers. Upon closer examination, the case of the Spurious Drugs Kingpin thus appears to have been more about mobilizing a language of public safety in order to police the profits of pharmaceuticals, and less about the quality of these pharmaceuticals themselves.

## The public in public safety

This tale of a Spurious Drugs Kingpin, idiosyncratic policing and ambiguous pharmaceuticals was itself ultimately unresolved in the courts. What it did clearly produce was a corollary story of mounting public anxiety. As one newspaper reported: ‘Over the past couple of weeks, enough has been going on in Chennai to get its residents a little worried about whether they are getting genuine, quality drugs when they are going to a pharmacy’ (‘Drug Case Suspect Held,’ 2010). The spectre of an unsafe drugs supply spooked many in the city and appeared to override the otherwise well-practiced social and economic divisions. Yet the feeling of urgency in response to a perceived public health danger itself poses the question: Who was collected in this collective?

This story of spurious drugs was on my radar as I, too, was a Chennai resident in 2009 and 2010 and read the city’s newspapers that seemed to carry new revelations about the case on the front pages of most days’ editions. During the early summer months of 2010, I spent a lot of time driving around the city. One morning, as I tuned my car radio to one of the two or three FM stations that employed chirpy young men and women disc jockeys to play the latest hits from Tamil cinema, the DJ’s atypically worried voice caught my ear. In Tamil, she asked many times over the course of her show, urging people to phone in, ‘*Namma Chennaikkarangalakku, yenge ponalum, yenna pannalum, poli marunthu pirachenaippathi pesikitterirukkom! Ithu oonmaiya? Poyya? Yarukku teriyum? Aiyo aiyo, nammalakku payam! Ungallukku enna abipriyam?*’ [Chennai-ites! Wherever we go, whatever we do, we are all talking about this drugs scandal! Is it true? Is it just a rumour? Who actually knows? We are all scared! What’s your view?]

Such talk made a change from the standard radio repartee. Until then, this DJ’s most incisive observations had been confined to debating listeners over which of Chennai’s cricket celebrities deserved the title of Number One Heart-Throb. As the mercury started to climb in the hot season, so, it seemed, did anxiety levels. Somehow, the everyday practices of local criminality – the existence of which was regularly acknowledged but something that middle-class people rarely expected to experience directly – had spilled over to threaten the affluent Chennai whom the station targeted as its audience.

This was a populace who regularly consumed – or at least aspired to consume – the best health that money can buy. Indeed, the city is home to Apollo Hospitals, India’s first (and in 2010 arguably still the most prestigious) ‘corporate’ hospital chain (Hodges, 2016). ‘We can’t trust drugs anywhere – *even at Apollo*!?’ came friends’ worried question, as we caught up over evening drinks on the city’s rooftop verandas., Their questions rehearsed the radio DJ’s fears: was no one safe when it came to Indian drugs? Although the middle classes were alarmed by this episode, this was very much a case where, if indeed the drugs in question presented a clear and present danger to health, the victims were most likely to be the city’s wage-labouring poor, not the salaried rich.

Why? First, the question of *which* drugs were allegedly being relabelled bears some investigation. Recall the death of a three-year-old – purportedly due to antibiotics that her parents bought from their local pharmacy in a poor part of town. Yet antibiotics, although they are prescribed and consumed across Chennai’s socio-economic gamut, played no role in the case of the Spurious Drugs Kingpin. Instead, the relabelled products that the police seized were products such as liver tonics for tiredness, pain killers and anti-rheumatics for achy joints, vitamin and mineral supplements, and cough syrups (‘Rs 1 Cr Worth,’ 2010; Vannan, 2010a). These are taken to ameliorate the symptoms of chronic conditions. They mask the structural aches and pains at the heart of what it is to belong to the city’s working poor, who do their utmost to carry on going to work, regardless of feeling run down or exhausted. And I use the term ‘working poor’ here advisedly; it is they who buy these drugs. The destitute cannot afford them.

Similarly, the working poor’s daily budgets rarely allow them to buy drugs to keep on hand; chemists regularly accommodate customers’ requests to sell single doses by taking a pair of scissors and cutting a tablet in its blister pack from a larger foil strip (‘Sell Drugs in Strips’, 2010). In effect, whatever the intent, pharmacists fulfilling these requests means that these customers would find it nearly impossible to check drugs’ expiry dates.

Both the radio DJs and my friends were clear: they were concerned about spurious drugs posing a threat to their personal safety. How could one safeguard one’s own health, they asked, if medicine, a key building block of health, is suspect? But I continued to wonder: what connected my well-heeled friends in the city’s leafy southern suburbs to the site of the initial crime – a north Chennai neighbourhood almost always preceded by the adjective ‘notorious’, and the only place I have ever had an auto-rickshaw driver refuse to take me, for fear of his own safety?

A fake drugs scandal could arguably prompt a trenchant review of enduring global inequalities. Yet, in the case of the Spurious Drugs Kingpin, the poor constantly receded from view. And the middle classes appear to have misrecognised dangers to the poor as dangers to themselves. Why? The measure of their anxiety struck me as much larger than seemed warranted. What might account for this misrecognition? Perhaps Chennai’s affluent residents recognized their own spurious drugs scandal as part and parcel of a different story: the scourge of global pharmaceutical counterfeiting.

## The address of global health

At this point, it is useful to pull back the focus of our story, and consider this question of middle class misrecognition against a different scale of inquiry. For some time, India’s media horizon has been awash with alarming stories about fake drugs (Quet, 2016). Headlines have included ‘New counterfeiting report highlights worrying trends’ (Barnes, 2007), ‘Fake drug industry operates openly’ (Shrivavasta & Narayana Kumar, 2007), ‘Most fake drugs pass every test’ (Shrivastava, 2007), ‘India becomes a hub for fake medicines’ (Lakshmi, 2010), ‘The fake drug industry is exploding and we can’t do anything about it’ (Ossola, 2015), ‘Indians at higher risk of getting fake drugs’ (Chandna, 2015), ‘Pills that kill’ (Annuncio et al., 2003). Most of these articles are in the popular press and fail to cite research. Even the World Health Organization has long pointed out the difficulty in getting accurate estimates about fake drugs: ‘information on the scale of the problem is inadequate and there are no global studies conducted’ (WHO, 1999, p. 3).

Indeed, global norm-setting institutions have fallen short of providing clear guidance. Over the past few decades, the world has witnessed an explosion of worry about fake drugs. Worries have poured forth from global health researchers, policy makers, states, nongovernmental organizations, health care workers, journalists, and, of course, consumers. All have raised the collective alarm about the urgent and life-threatening dangers that these drugs potentially present. During these decades, norm-setting bodies, such as the World Health Organization and World Trade Organization, have found themselves in the middle of controversy when framing definitions of different kinds of non-normative pharmaceuticals: ‘counterfeit’, ‘expired’, ‘substandard’, ‘spurious’, and so on. For individual consumers, however, the questions tend to be simpler. We ask ourselves: Will this make me better? Is it safe?

Perhaps surprisingly given the relative paucity of hard data, research publications have also followed this trend and regularly highlight India as a place where the drugs supply is of particular concern. Some authors report that:

Over the past decade, the number of countries reporting falsified (fake, spuriously/falsely labelled/counterfeit) medicines and the types and quantities of fraudulent drugs being distributed have increased greatly. The obstacles in combatting falsified pharmaceuticals include … deficient regulation and regulatory challenges, especially in China and India where fake products often originate. (Nayyar et al., 2015, p. 113)

Another article in the *Lancet* announced: ‘The market in fake and substandard pharmaceuticals is not unique to India. But one widely quoted WHO statistic places this country as the leader, with as much as 35% of the world’s production’ (Chatterjee, 2001, p. 177). Nevertheless, the few studies that exist have struggled to produce significant evidence to substantiate these claims. Others have suggested that the problem is not on a comparable scale to the rhetoric employed.

Anxiety about fake drugs produces a particular form of public health scandal, and rehearses a set of spectacular forms: the perp walk, the drug bust, the worried chat. These accounts of Indian pharma’s twenty-first century history are saturated with stories from the coterminous rise of the global anti-counterfeiting movement (Hornberger, 2018). They are coupled with expressions of worry manifest in the quotes and headlines above, repeated over and over again by both expert and popular outlets. Together, they suggest that to question the scourge that fake drugs present seems absurd, the doubt itself dangerous. Yet in the case of the Spurious Drugs Kingpin and his alleged empire of expiry, questions of where danger actually lies and where blame should fall are far from clear.

The ambivalence of affluent India’s ‘arrival’ also haunts middle-class Indians’ peculiar engagement with this particular global public health imaginary. Through it, the Kingpin’s alleged practices – and the enduring ambiguity of the danger/safety of expired energy tonics – transmogrify into the object of global anti-counterfeiting surveillance, and the worries of the middle class toggle between a misrecognition of their common cause with those from the notorious north Chennai neighbourhood, Pulianthope, and an over-identification with the global elite who consume news about the plethora of fake pharmaceuticals. They further merge with a well-practiced distrust of the Indian state, a concern that the state fails to protect the interests of its citizenry, which is shared by many Indians.

Alongside the popular circulation of international law lies a longer history of suspicion held by Indians regarding the veracity of consumer commodities and trickster figures. Indeed the Hindi slang for a fraudster was immortalised in the name of a popular Bollywood film – *Shree* 420. 1955. Dir Raj Kapoor in which the eponymous character is named in homage to Section 420 of the Indian Penal Code: the law for fraudsters. This cultural familiarity with and expectation of copies, duplicates, and fakes has been commented on extensively as part and parcel of a (post)colonial engagement with modernity (see, e.g. Abbas, 2008; Bhabha, 1984; Wong, 2017). After 1991 and the loosening of import duties in the wake of the nation’s programme of structural adjustments and trade liberalization, Indians both enjoyed more imported goods, including imported copies. Thus, broader experiences of consumption brought along in its wake a creeping suspicion about precisely these new consumer opportunities. In a similar vein, the case of the Spurious Drugs Kingpin represented the betrayal of the social contract that is at the heart of any commercial pharmaceutical transaction. The case of the Spurious Drugs Kingpin bore a family resemblance to this longer history of copies and suspicion of purveyors. The middle-class call and response of Indian broadsheet journalists and their audiences arguably made sense of this betrayal of the social contract in precisely such terms.

In the story of the Spurious Drugs Kingpin, the middle classes’ misrecognition of their own danger is a function of the merging with the local landscape of drugs circulation in Chennai with the broader, if more abstract, landscape of ambivalence within otherwise urgent public health messaged. Anxieties about drugs and danger of global health emerge as a character in this Chennai story, as city residents struggle to make sense of the seemingly shocking and amoral set of alleged activities at the heart of the story: the retail recirculation of relabelled expired drugs. In trying to make sense of this, both middle-class journalists and their middle-class readers disregard questions of their own unlikely proximity to cough syrup and energy tonics, let alone pharmacies in notorious neighbourhoods of the city’s labouring classes. The apparent take-away lesson is harder to swallow: the policing of drugs is not in service of public safety, but rather is evidence that the city’s police act at the behest of pharmaceutical manufacturers. What the middle-class journalists and readers mobilise to make sense of this episode is an already-circulating pharmaceutical trope: the always-suspected counterfeit. What appears to be more the case is a garden variety case of piracy: when distributors become manufacturers.

Of piracy, Dent (2016) suggests that when distributors become authors (or, in this case, manufacturers) troubles start. And indeed it is precisely the doubling of distributors into authors of drugs that produced the scandal under consideration. Insofar as this scandal has a resolution, what seems to be put back into place is the ability of manufacturers to control both the authorship of, and with it the profit accruing to, their product. What is completely absent in this resolution, however, is the matter of drug safety. In the place of public health certainties, we must confront the ambiguity of the expired drugs at the heart of the story.

But this ambiguity is itself productive. Returning to the questions posed by the story of the kingpin, what does it matter that the Indian middle-class consumer ventriloquizes global health discourse about the dangers of fake drugs? The ambiguous safety status of expired, redistributed drugs provides an opening for misrecognition; the misrecognition is itself a resolution. For health matters as considered among affluent Chennai-ites, the mis-recognition of the Spurious Drugs Kingpin as a player in the duplicitous global market in health precipitates a dreadful question, itself impossible for drawing clear lines or achievable outcomes: ‘In India, even if we are rich, are we the haves? Or, are we still the have-nots?’

## Notes

1. In real terms quite a lot, but hardly a king’s ransom. At that point in time, the sum might have bought you two decent used cars.

2. Around $20 million in 2010.

3. This was an income tax charge: V. Meenakshisundaram, Chennai vs Department of Income Tax on 19 August 2010. In this case, heard before the Income Tax Tribunal, Meenakshi Sundaram admitted that he was using two properties for his business. The tribunal held that he could not classify them as ‘income from house property’ head, when he was clearly using it for business purposes.

4. Vasantha Meena Enterprises vs Baskaran on 6 June 2011.

5. M/s. Meena Health Care (P) Ltd., Rep. By Its Managing Director V. Meenakshisundaram Vs the State.

6. V. Meenakshisundaram (MS) vs The Inspector of Police on 18 August 2010.
